# Clinical Characteristics and Management of Patients With Clinical Amyopathic Dermatomyositis: A Retrospective Study of 64 Patients at a Tertiary Dermatology Department

**DOI:** 10.3389/fmed.2021.783416

**Published:** 2021-12-02

**Authors:** Keyun Tang, Hanlin Zhang, Hongzhong Jin

**Affiliations:** Department of Dermatology, State Key Laboratory of Complex Severe and Rare Diseases, Peking Union Medical College Hospital, Chinese Academy of Medical Science and Peking Union Medical College, National Clinical Research Center for Dermatologic and Immunologic Diseases, Beijing, China

**Keywords:** amyopathic dermatomyositis, clinical characteristics, treatment, 2017 EULAR/ACR classification criteria, antimalarials, diagnosis

## Abstract

**Background:** Clinical amyopathic dermatomyositis (CADM) represents a subtype of 5–20% of patients with dermatomyositis (DM), which can be categorized into amyopathic dermatomyositis (ADM) and hypomyopathic dermatomyositis (HDM). The characteristics of patients with CADM are still limited in English literature.

**Objective:** To investigate clinical features, cutaneous findings, diagnostic accuracy, and treatment regimen of CADM patients.

**Methods:** Sixty-four patients diagnosed with CADM at Peking Union Medical College Hospital by dermatologists were retrospectively analyzed. Data were recorded in the electronic database at each offline clinical consultation and directly extracted from medical records. 2017 EULAR/ACR criteria for idiopathic inflammatory myositis (IIM) classification was used to identify and classify patients with CADM. Published studies were searched to extract relevant data of CADM patients.

**Results:** This cohort included 38 ADM patients and 26 HDM patients. 2017 EULAR/ACR criteria classified 67.2% of patients with CADM into probable or definite DM. Antimalarials were given to a majority of CADM patients (72.6%, *n* = 45). However, 68.8% (31 out of 45) required at least one aggressive agent combined with hydroxychloroquine due to insufficient response or side effects. The median of systemic treatments in HDM was significantly higher than ADM (*p* = 0.007). The number of ADM patients using antimalarials as monotherapy was significantly higher than that of HDM patients (*p* = 0.031), while the number of HDM patients receiving steroids combined with immunosuppressants was significantly higher (*p* = 0.025). The median of Cutaneous Dermatomyositis Disease Area and Severity Index (CDASI) score improvement was 11.5 and 10.5 for ADM and HDM after a median follow-up of 31.5 and 32.5 months, respectively. Six patients with normal muscle strength developed muscle weakness after a median of 10.5 months (IQR 9-13), and elevated inflammatory markers at initial visit might indicate their muscle weakness development.

**Conclusions:** 32.8% of patients may be overlooked using the three skin variables of 2017 EULAR/ACR criteria. The response rate to single hydroxychloroquine in our cohort was 68.8%. Detailed treatment modalities were different among ADM and HDM. Long-term monitoring for the development of myositis in patients with CADM, especially those with elevated inflammatory markers at initial visit, may be warranted.

## Introduction

Clinical amyopathic dermatomyositis (CADM) is defined as occurrence of the hallmark cutaneous manifestations of dermatomyositis (DM) without muscle weakness for 6 months or longer (1, 2), which accounts for at least 20% of all DM (3). The concept of ADM as a subcategory of inflammatory idiopathic myositis (IIM) was initially proposed by Euwer and Sontheimer in 1991 (4). However, the diagnosis and classification of CADM have not been well-recognized by most existing criteria for DM in contrast to developments of categorization of classic DM (CDM) and other subgroups.

The definite diagnosis for CADM remains a problem for clinicians for years. In 2002, Sontheimer proposed the first and only standalone classification criteria for ADM (5). In 2003, Dalakas and Hohlfeld came up with new criteria of IIMs and included sine DM/ADM as a subgroup of IIMs (6). In 2004, Hoogendijk et al. further solidified the entity of ADM as a subset of the spectrum of IIMs at the 119th European Neuromuscular Center international workshop (7). In 2017, the EULAR/ACR jointly proposed new classification criteria for adult and juvenile IIM and their major subgroups (8). Although some studies investigated the performance of the EULAR/ACR IIM criteria in diagnosed IIM cases (9–11), its applicability in the group of CADM is still limited. In addition, although cutaneous symptoms represent a significant burden for DM patients, the therapeutic approach of CADM have not been well-recognized compared to classic DM.

This study set out to expand our knowledge of CADM. The performance of EULAR/ACR classification criteria in Asian CADM populations was validated for the first time, and specific medications and treatment regimens of ADM and HDM were also investigated. The comparative data of CADM patients from other clinical series were analyzed to understand CADM more comprehensively and thoroughly.

## Methods

### Patient Population

This is a retrospective study of patients with the diagnosis of CADM seen at Peking Union Medical College Hospital (PUMCH) by a dermatologist between October 1, 2012 to December 31, 2018. The institutional board of Peking Union Medical College Hospital approved the data collection of clinical information (No. S-K1679). Patients were at least 18 years old at enrollment. The diagnosis of CADM was determined by the main investigator using criteria proposed by Sontheimer and Gerami and ultimately confirmed by a senior dermatologist (Dr. HZ Jin) (12, 13) (Supplementary Table 1). Patients who had a suspicious diagnosis of CADM, irregular visits and skin evaluation at our center (>6 months between two visits in their first 2 years of follow-up), total follow-up period <1 year, or loss of follow-up were excluded from analysis (*n* = 12). CADM patients presented with typical cutaneous manifestations of DM (heliotrope rash, Gottron's sign, and Gottron's papules) with no evidence of muscle weakness for at least 6 months after the first consultation. Patients with CADM at baseline were further classified into hypomyopathic DM (HDM), if they had subclinical evidence of myositis (abnormalities in muscle enzyme, electromyography (EMG), or muscle biopsy or MRI), otherwise, amyopathic DM (ADM) (13). CADM patients developing muscle weakness during 6 months follow-up were categorized as CADM → classical DM (13). Thus, 64 patients were included and were further categorized into 38 ADM and 26 HDM. Among 38 ADM patients, 25 had no abnormities in extensive muscle testing (EMG and muscle biopsy). The results of EMG or biopsy of other 13 patients was unavailable, but they fulfilled other definitions of ADM and was also included in the final analysis. According to the documented muscle testing of ADM and HDM patients (*n* = 46), the negative predictive value of muscle enzymes was 86.2% (Supplementary Table 2), which supports that the classification of ADM patients without extensive muscle testing that may well belong to ADM (14).

Cutaneous Dermatomyositis Disease Area and Severity Index (CDASI) were applied by the same dermatologist to evaluate cutaneous findings of patients at each follow-up examination. The information was prospectively collected into a database by the same dermatologist at each outpatient consultation. CDASI included the type of skin disease and 15 anatomical locations. Gottron's signs and papules, periungual changes, and alopecia were also scored by CDASI. CDASI of 5 or less was defined as complete remission of skin disease (15). CDASI of each patient was obtained at ~3–6 months within their first 2 years of follow-up.

### Data Collection

We extracted the following data if available: demographics, cutaneous manifestations, muscle strength, follow-up period, muscle enzymes, erythrocyte sedimentary rate (ESR), C-reactive protein (CRP), myositis-specific autoantibodies (MSA), antinuclear antibodies (ANA), EMG, skin biopsy, muscle biopsy, MRI, high-resolution computed tomography (HRCT), and concomitant diseases, such as malignancy and interstitial lung disease (ILD). If information was partially missed in their medical records, the patients were inquired at the next follow-up visit, or directly contacted via telephone and online chatting.

The MSA profiles were assessed using a EUROIMMUN AG kit by an immunoblotting assay, which included 16 anti-autoimmune inflammatory myopathy Ags. anti-melanoma differentiated-associated protein 5 (anti-MDA-5), anti-Mi-2α, anti-Mi-β, anti-TIF1γ, anti-NXP2, anti-SAE1, anti-signal recognition particle (SRP), anti-RO-52, anti-PM-SCL-75, anti-PM-SCL100, anti-Ku, anti-Jo-1, anti-threonyl-tRNA synthetase (anti-PL7), anti-alanyl-tRNA synthetase (anti-PL12), anti-glycyl tRNA synthetase (anti-EJ), and anti-isoleucyl-tRNA synthetase (anti-OJ), were included in the MSA profiles.

According to the 2017 EULAR/ACR criteria for inflammatory idiopathic myositis, we scored using an online calculator webpage (www.imm.ki.se/biostatistics/calculators/iim). The score of probable IIM is no <5.5, or 6.7 when muscle biopsies were performed (corresponding to a cutoff probability of 55%). The performance of the 2017 criteria was investigated in classifying CADM.

Since our department is a tertiary dermatology center, the majority of our patients have been treated before referrals. In our study, systemic treatments included oral or intravenous corticosteroids, hydroxychloroquine, immunosuppressants (cyclophosphamide, CTX; methotrexate, MTX; mycophenolate mofetil, MMF; cyclosporine A, CsA; tacrolimus), thalidomide and intravenous immunoglobin (IVIG). Topic treatments refer to topical corticosteroids and topic calcineurin inhibitors (tacrolimus, pimecrolimus).

### Literature Review

A literature review search was conducted on PubMed database up to November 3rd, 2021 to find the relevant studies on CADM with the following search strategy: amyopathic dermatomyositis[Title/Abstract] OR hypomyopathic dermatomyositis[Title/Abstract]. Original research written in English, published in the recent 15 years (from 2006 to 2021), with sample size of adult-onset CADM no <5, were included. The data of CADM patients could be separated from a large series (e.g., IIM, or DM/PM). These studies should focus on clinical features of CADM rather than epidemiologic data or a specific disease (e.g., ILD or malignancy). We extracted clinical information of these studies if available, including the name of the first author, publication year, location of study, sample size, study type, diagnostic criteria, demographics, skin findings, examinations, treatments and other comparative data with those of our series.

### Statistical Analysis

Qualitative data are presented as frequencies and percentages. Quantitative data are presented as means ± standard deviations or medians (ranges). Qualitative data such as treatment, remission, and laboratory findings were analyzed with the Fisher's exact test χ^2^ test or as appropriate. Quantitative data, including CDASI score and follow-up time of the patients, were analyzed by the Mann-Whitney test. Statistical analysis was conducted using SPSS (Version 25.0, IBM SPSS Statistics). A *p* <0.05 was considered statistically significant. Binary logistic regression models were used to identify risk factors associated with cancer, ILD and muscle weakness development for CADM patients.

## Results

### Clinical Information of Patients in PUMCH

#### Demographics and Clinical Findings

Sixty-four patients were included in the final analysis (Table 1). The majority of patients were Asian females (75%), with a mean age of 45.3 years at the time of enrollment. The subgroup of CADM included 59.3% with ADM and 40.7% with HDM. The mean age at DM diagnosis was 42.1 years for ADM patients and 50.1 years for HDM patients, respectively (*p* = 0.046). The diagnosis of HDM was verified by a combination of positive findings of muscle enzymes (22/26, 84.6%), EMG (10/21, 47.6%), and MRI (12/14, 85.7%). 31.6% of ADM patients (*n* = 12) and 53.8% of HDM patients (*n* = 14) tested positive for ANA.

**Table 1:** Demographics and clinical findings of 64 included patients with clinical amyopathic dermatomyositis.

	**ADM**	**HDM**	**CADM**	***p*-value**
Patients (*n*/%)	38 (59.3)	26 (40.7)	64 (100)	
**Demographics**
Female sex	29 (76.3)	19 (73.1)	48 (75)	1.000
Age at disease diagnosis (years)	42.1 ± 15.1	50.1 ± 16.3	45.3 ± 16.0	**0.046**
Time to diagnosis (mons)	5.7 ± 6.5	6.3 ± 6.9	5.9 ± 6.6	0.541
**Examinations**
Elevated serum muscle enzymes (CK, LDH, AST)	0/38 (0)	22/26 (84.6)	22/64 (34.4)	**<0.001**
Myositis in extensive muscle testing (EMG and muscle biopsy)	0/25 (0)	10/21 (47.6)	10/46 (21.7)	**<0.001**
Elevated Inflammatory markers (ESR, hsCRP)	3/38 (7.9)	6/26 (23.1)	9/64 (14.1)	0.142
Positive ANA (≥1:80)	12/38 (31.6)	14/26 (53.8)	26/64 (40.6)	0.119
Positive MSA	7/8 (87.5)	6/6 (100)	13/14 (92.9)	1.000
Muscle inflammation on MRI	0/18 (0)	12/14 (85.7)	12/32 (37.5)	**<0.001**
**Concomitant diseases**
Interstitial lung disease	15 (39.5)	13 (50)	28 (43.8)	0.450
Malignancy	3 (7.9)	5 (19.2)	8 (12.5)	0.467
**Treatments**				
Median of systemic treatments	1.5 (1–2)	2 (1.75–3)	2 (1–2)	**0.007**
Monotherapy	17 (44.7)	6 (23.1)	23 (35.9)	0.112
Oral steroids	2 (5.3)	4 (15.4)	6 (9.4)	0.213
Antimalarials	12 (31.6)	2 (7.7)	14 (21.9)	**0.031**
Immunosuppressants	3 (7.9)	0 (0)	3 (4.7)	0.265
Combined therapy	21 (55.3)	20 (76.9)	41 (64.1)	0.112
Steroids + antimalarials	9 (23.7)	4 (15.4)	13 (20.3)	0.534
Steroids + immunosuppressant	2 (5.3)	7 (26.9)	9 (14.1)	**0.025**
Antimalarials + immunosuppressant	4 (10.5)	0 (0)	4 (6.3)	0.140
Antimalarials + immunosuppressant + thalidomide	2 (5.3)	2 (7.7)	4 (6.3)	1.000
Steroids + antimalarials + immunosuppressant	4 (10.5)	6 (23.1)	10 (15.6)	0.293
Steroids + antimalarials + immunosuppressant + IVIG	1 (2.6)	1 (3.8)	2 (3.1)	1.000
**In total**
Steroids	18 (39.1)	24 (92.3)	42 (58.3)	**<0.001**
Antimalarials	32 (88.9)	13 (50)	45 (72.6)	**0.001**
Immunosuppressants	16 (42.1)	16 (61.5)	32 (50)	0.203
Thalidomide	2 (5.3)	2 (7.7)	4 (6.3)	1.000
Topical treatments	35 (92.1)	24 (92.3)	59 (92.2)	1.000
**Outcomes**
Median follow-up (mons)	31.5 (21.75–58)	32.5 (26.75–57.5)	32 (24–56.75)	0.312
Clinical remission of skin disease	25 (65.8)	21 (80.8)	46 (71.9)	0.261

*Bold value means P <0.05*.

A total of 28 patients (43.8%) were diagnosed with ILD based on patient history, clinical symptoms and HRCT findings. Eight cases of malignancy (12.5%) developed among the 64 CADM patients. Lung cancer accounted for 50% (*n* = 4) of malignancies and is the most common type in our cohort, followed by breast cancer (*n* = 3) and thyroid cancer (*n* = 1). The incidence of concomitant diseases was similar between ADM and HDM group.

#### Skin Evaluation and Disease Course

The median CDASI score at the initial visit was 16.5 (13–21.25) and 17.5 (14–23.25) for ADM and HDM, respectively (*p* = 0.389) (Table 2). The score improvement during follow-up was 11.5 (9–17) and 10.5 (8.25–17.25) for these two groups after a median follow-up month of 31.5 (21.75–58) and 32.5 (26.75–57.5), respectively. At the last visit, the three most common areas of cutaneous findings were periorbital (44.7%), rest of face (44.7%), and periungual skin (34.2%) in ADM, instead, periorbital (38.5%), v-area of neck (38.5), and rest of face (30.8%) in HDM.

**Table 2:** Cutaneous findings of patients with dermatomyositis at baseline visit as measured by CDASI activity and damage.

	**At initial visit**		
	**ADM**	**HDM**	***p*-value**
**CDASI score, median (IQR)**			
Total	16.5 (13–21.25)	14.5 (10.75–21.00)	0.182
CDASI activity-score	15 (12–21)	12.5 (9–19)	0.164
CDASI damage-score	1.5 (1–2.25)	1 (1–2)	0.457
Score improvement	11.5 (9–17)	10.5 (8.25–17.25)	0.686
**Cutaneous finding**			
Periorbital	30 (78.9)	18 (73.1)	0.765
Gottron's hands	21 (55.3)	12 (50.0)	0.800
Gottron's not on hands	16 (42.1)	10 (38.5)	0.801
Scalp	7 (18.4)	5 (19.2)	1.000
Malar area	13 (34.2)	11 (42.3)	0.602
Rest of face	28 (73.7)	19 (76.0)	1.000
V-area of neck	24 (63.2)	17 (65.4)	1.000
Posterior neck	15 (39.5)	10 (35.7)	0.802
Upper back and shoulders	21 (55.3)	14 (53.8)	0.804
Rest of back and buttocks	11 (28.9)	7 (26.9)	1.000
Abdomen	5 (13.2)	9 (34.6)	0.064
Lateral upper thigh	12 (31.6)	6 (23.1)	0.575
Rest of leg and feet	10 (26.3)	13 (50.0)	0.067
Arm	16 (42.1)	15 (57.7)	0.309
Dorsum of hands	6 (15.8)	4 (15.4)	1.000
Alopecia	2 (5.3)	0 (0)	0.510
Periungual changes	18 (47.4)	10 (38.5)	0.609
Mechanic hands	14 (36.8)	6 (23.1)	0.283

**Figure 1:**
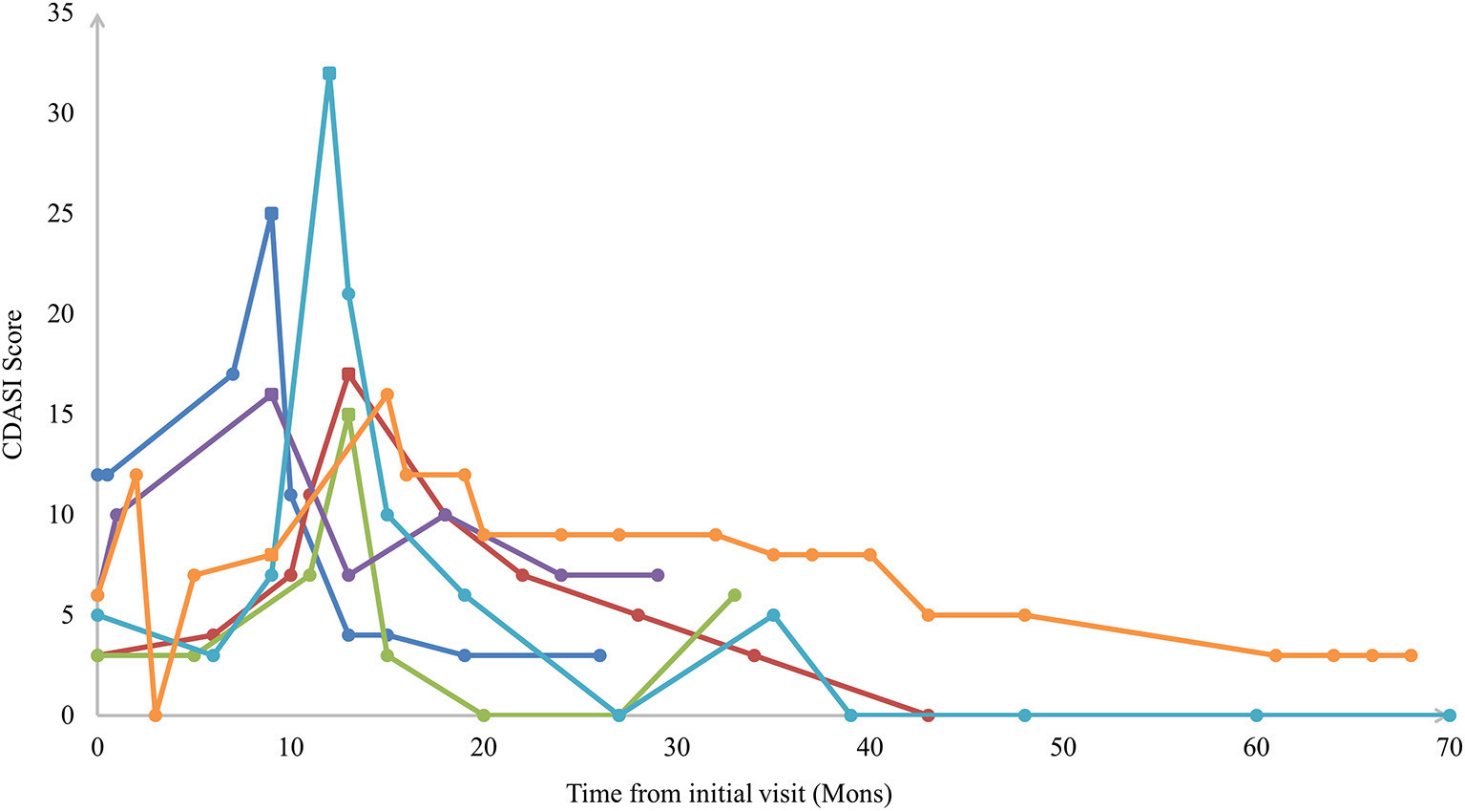
Sample plots of Cutaneous Dermatomyositis Assessment and Disease Severity (CDASI) among patients with extended clinical amyopathic dermatomyositis vs. follow-up time. The square icon indicated the CDASI score at the time of developing clinical muscle weakness.

Fifty-eight CADM patients had normal muscle strength constantly while 6 HDM patients developed muscle weakness (CADM → classical DM) after a median of 10.5 months (IQR 9-13). The CDASI score peaked as the same time of developing clinical muscle weakness in 5/6 of the patients (Figure 1). The other 1 patient developed muscle weakness at 9 months after the initial visit with a CDASI score of 8. Her peaked CDASI score was 16 occurring at month 15 after the initial visit, and muscle strength of upper and lower limbs was grade 4 in month 15 (Supplementary Table 3). Univariate regression analysis showed that the elevated Inflammatory markers at initial visit may be associated with muscle weakness development in CADM patients (Supplementary Table 4).

#### Diagnosis

73.4% of patients (*n* = 41) were misdiagnosed with other diseases before referral to our center, such as allergic dermatitis (*n* = 12), seborrheic dermatitis (*n* = 9), eczema (*n* = 8), discoid lupus erythematosus (*n* = 4), systematic lupus erythematosus (*n* = 2), undifferentiated connective tissue disease (*n* = 2), drug eruption (*n* = 2), and lichen planus (*n* = 2). The remaining misdiagnosis included erythema multiforme, pityriasis rubra pilaris, Adult-onset Still disease, cutaneous vasculitis, rosacea, and alopecia areata.

2017 EULAR/ACR IIM criteria yielded a sensitivity of 67.2% in our CADM patients with a cutoff value of 55% (Supplementary Table 5). Among the 6 excluded HDM patients, 1 only had heliotrope rash, and 2 only had Gottron's sign. The other 3 did not present these three skin variables at the initial presentation. Among the 13 excluded ADM patients, 9 only had heliotrope rash, 2 only had Gottron's papules on hands, and 1 had Gotton's sign on knees. The other 1 without any characteristic skin manifestations were suspected to have DM pathologically and developed Gottron's sign on his elbows 3 months after the initial visit. Despite the three variables, common cutaneous findings in this group were distributed in the rest of the face (15/19), upper back and shoulders (13/19, V-area of neck (11/19), and arms (9/19).

We further analyzed the presence of the three skin variables of 2017 EULAR/ACR criteria (heliotrope rash, Gottron's papules, and Gottron's sign) in 64 CADM patients at the time of enrollment (Supplementary Table 6). 18.7% of patients (*n* = 12) had all three characteristic skin findings. 42.2% (*n* = 27) of patients presented with two out of the three skin variables and 32.8% (*n* = 21) of patients had only one skin variable. Four patients who had none of the three skin presentations developed typical DM rash later in the follow-up.

#### Treatments

The median of systemic treatments for whole cohort was 2 (IQR: 1–2), and the number of systemic agents given to HDM was significantly higher than that of ADM patients (*p* = 0.007) (Table 1). The number of ADM patients using antimalarials as monotherapy was significantly higher than that of HDM patients (*p* = 0.031), while the number of HDM patients receiving steroids combined with immunosuppressants was significantly higher (*p* = 0.025). Of 38 ADM patients, three only used immunosuppressive agents (MTX: *n* = 2; MMF: *n* = 1). As for combined therapy, immunosuppressants included MTX (*n* = 5), MMF (*n* = 3), CsA (*n* = 2), Tac (*n* = 2), and CTX (*n* = 1). HDM patients used MTX (*n* = 8), Tac (*n* = 5), CTX (*n* = 3), and MMF (*n* = 2). The proportion of ADM patients treated with antimalarials was significantly higher than that of HDM (*p* = 0.001, 88.9 vs. 50%). The proportion of steroids applied in HDM group was significantly higher (*p* <0.001, 92.3 vs. 39.1%).

In total, 45 out of 64 patients (72.6%) with CADM received antimalarials at some stages of disease course. Of these 45 patients, 31 (68.8%) were treated with at least one conjunctive agent for adequate disease control. Of the entire cohort, 50% (32 out of 64) needed at least one immunosuppressant to achieve control of skin symptoms. MTX (*n* = 15, 23.4%) and Tac (*n* = 5, 7.8%) are the most commonly used immunosuppressive agents. After a median of 32 months follow-up, 71.9% of patients (*n* = 46) achieved clinical remission of skin disease.

### Clinical Information of Patients in Previous Studies

A total of 906 CADM cases in 30 clinical studies were included in this analysis (Table 3). Most of studies were conducted in China (*n* = 9, sample size = 336), followed by America (*n* = 8, sample size = 420) and Japan (*n* = 7, sample size = 82). Among these, 136 were specified as ADM and 36 as HDM, and the other 734 were classified as CADM. The average age at disease diagnosis ranged from 41.8 to 69.4 years in different series. Females accounted for 40–96.6% with a total proportion of 76.4% (593/776). Based on the available data of skin findings, The proportion of heliotrope rash, Gottron's papules and Gottron's sign were 59.6% (270/453), 57.3% (177/309), and 80.6% (179/222), respectively. The descriptions of examinations were limited. One hundred and thirteen out of 263 patients (43.0%) testing for ANA were positive. In extensive muscle testing, myositis was indicated in 25.5% (13/51) and 15.2% (7/46) of patients by EMG and muscle biopsy, respectively. ILD and malignancy were two predominant concomitant diseases among CADM. 61.3% (301/493) of patients were complicated by ILD (range: 0–80.6%), while malignancy was reported in 13.9% (36/259, range: 0–85.7%).

**Table 3:** Clinical information of patients with clinically amyopathic dermatomyositis from previous studies.

**First authors**	**Location**	**Sample size (n)**	**Study type**	**Diagnostic criteria**	**Journal**	**Age at diagnosis (years)**	**Female sex (*n*, %)**	**Skin findings**		**Examinations (*n*, %)**	**Concomitant diseases (*n*, %)**	**Treatments**
								**Typical skin rash of DM (*n*, %)**	**Other characteristic skin rash (*n*, %)**			
Rodríguez-Tejero (24)	Spain	5	Case series	NA	Dermatolo Ther	61.6 ± 12.6	2 (40)	Heliotrope rash: 2 (40) Gottron's papules: 3 (60) Gottron's sign: 2 (40)	V-neck sign: 2 (40) Shawl sign: 2 (40)	NA	ILD: 0 (0) Malignancy: 2 (40)	Topical agents: 5 (100) Systemic treatments: 4 (HCQ: 2, HCQ + MMF: 1, MTX + IVIg: 1)
Zhu (31)	China	41	Case series	Sontheimer criteria	Rheumatology (Oxford)	52.7 ± 1.78	31 (75.6)	Heliotrope rash: 23 (56.1) Gottron's papules: 19 (46.3) Gottron's sign: 22 (53.7)	V-neck sign: 11 (26.8) Shawl sign: 3 (7.3) Mechanic hands: 6 (14.6)	NA	ILD: 34 (82.9)	NA
Kanaoka (32)	Japan	9	Case series	Sontheimer criteria	Rheumatology (Oxford)	64.3 ± 13.1	5 (55.6)	Heliotrope rash: 5 (55.6) Gottron's papules: 6 (66.7) Gottron's sign: 9 (100)	V-neck sign: 4 (44.4) Shawl sign: 3 (33.3)	NA	ILD: 4 (44.4) Malignancy: 1 (11.1)	NA
Bowerman (33)	America	59	Cohort study	Sontheimer criteria	J Am Acad Dermatol	53 (40–63)	57 (96.6)	NA	NA	NA	Malignancy in 2 years: 1/59 (1.7); 5 years: 2/37 (5.4)	NA
Gan (34)	China	108	Case series	Sontheimer criteria	Chin Med J (Engl)	50.4 ± 12.1, 49 ± 12.4	87 (80.6)	Heliotrope rash: 54 (50) Gottron's sign/papules: 87 (80.5)	V-neck sign and shawl sign: 57 (52.8) Mechanic hands: 41 (38)	NA	ILD: 87 (80.6); asymptomatic ILD: 22 (20.4)	NA
Shimizu (35)	Japan	7	Case series	Sontheimer criteria	J Dermatol	69.4 ± 12.7	4 (57.1)	All patients had a DM-specific skin eruption	NA	All with detectable anti-TIF1-γ Ab	ILD: 1 (14.3) Malignancy: 6 (85.7)	Topical agents: 4 OS: 3 (20–30 mg/d)
Cassius (36)	Belgium	17 ADM	Case series	Sontheimer criteria	J Am Acad Dermatol	54 ± 14.7	12 (70)	NA	NA	NA	ILD: 5 (26.4) Malignancy: 1 (5.6)	NA
Pinard (18)	America	115 (93 ADM and 22 HDM)	Case series	ICD code	JAMA Dermatol	51.1 ± 14.6	105 (91.3)	NA	NA	NA	NA	Antimalarials: 88 (76.5); Needed more aggressive therapy: 78/88 (88.6) MTX: 59 (51.3); MMF or mycophenolic acid: 46 (40.0)
Borges (37)	Brazil	22	Single-center cross-sectional study	Sontheimer criteria	An Bras Dermatol	49.7 ± 14.7	12 (54.6)	Heliotrope rash: 19 (86.4) Gottron's papules: 21 (95.5)	V-neck sign: 10 (45.5) Shawl sign: 6 (27.3) Mechanic hands: 3 (13.6)	NA	ILD: 13 (59.1)	NA
Nishimi (38)	Japan	10	Case series	Sontheimer criteria	Clin Rheumatol	53.8 ± 3.7	6 (60)	NA	NA	NA	ILD: 10 (100)	NA
Patel (17)	America	110	Case series	Sontheimer criteria	J Am Acad Dermatol	DM+CADM: 51.9 ±13.3	DM+ CADM: 179 (84.8)	Heliotrope rash: 68 (61.8) Gottron's papules: 35 (31.8) Gottron's sign: 100 (90.1)	V-neck sign: 83 (75.5) Shawl sign: 65 (59.1) Mechanic hands: 66 (60)	NA	NA	NA
Peng (39)	China	20	Case-control study	Sontheimer criteria	Clin Rheumatol	49.2 ± 13.0 (age at symptom onset)	16 (80)	Heliotrope rash: 11 (55) Gottron's papules: 18 (90)	V-neck sign: 9 (45) Shawl sign: 6 (30)	ANA: 0/20 (0)	ILD: 17 (85)	NA
Li (40)	China	17	Case series	Sontheimer criteria	Clin Rheumatol	54.9	14 (82.4)	NA	NA	NA	NA	NA
George (41)	America	20	Case series	Sontheimer criteria	Br J Dermatol	58.2 ± 13.2	18 (90)	NA	Mechanic hands: 10 (50)	ANA: 6/18 (30)	ILD: 6/16 (38) Malignancy within 5 years: 2 (10)	NA
Xu (42)	China	40	Case series	ENMC workshop	Clin Rheumatol	53.6 ± 9.7, 48.8 ± 13.1 (age at symptom onset)	22 (55)	NA	Mechanic hands: 6 (15)	Elevated ESR: 14 (35) Elevated CRP: 10 (25) Positive ANA: 28 (70)	NA	NA
Galimberti (19)	America	44	Case series	Sontheimer criteria	Br J Dermatol	60 ± 13.1	40 (91)	Heliotrope rash: 18 (41) Gottron's papules: 29 (66)	Mechanic hands: 12 (27)	Positive ANA: 16/25 (64)	ILD: 5 (11.4) Malignancy within 5 years: 6 (13.6)	Topical steroids: 4 (3 improved) OS: 25 (20 improved and 22 needed more aggressive therapy) HCQ: 13 (5 improved and 8 needed more aggressive therapy), OS + HCQ: 1 (improved); OS + MTX: 1 (stable/worse)
Moghadam-Kia (43)	America	61	Case-control study	^**^	Arthritis Care Res (Hoboken)	44.8 ± 17.6	39 (64)	NA	NA	NA	ILD: 19 (31.1)	NA
Yamasaki (44)	Japan	5	Case series	Modified Sontheimer's definitions	Mod Rheumatol	51 ± 12	4 (80)	NA	NA	NA	ILD: 4 (80) malignancy: 0 (0)	NA
Gil (45)	Israel	5	Case series	NA	Clin Rheumatol	41.8 ± 17.7	NA	Gottron's papules: 3 (60) Gottron's sign: 2 (40)	V-neck sign: 2 (40)	Positive ANA: 3 (60) Elevated ESR: 2 (40) Elevated CRP: 1 (20)	ILD: 3 (60) Malignancy: 1 (20)	HCQ + OS + MTX + AZA + CTX + IVIG + MMF: 1 HCQ + OS + MTX + MP + IVIG + CTX: 1 MP + CTX + rituximab + plasma exchange: 1 HCQ + OS + MTX: 1 HCQ + MTX: 1
El-Dokla (46)	America	5 (HDM)	Case series	Sontheimer criteria	J Clin Neuromuscul Dis	54 ± 3.5	4 (80)	Heliotrope rash: 3 (60) Gottron's papules: 3 (60) Gottron's sign: 3 (60)	V-neck sign: 2 (40) Shawl sign: 2 (40) Mechanic hands: 1 (20)	Elevated muscle enzymes: 0 (0) Positive ANA: 4 (80), Myositis in EMG: 2 (40) Myositis in muscle biopsy: 5 (100)	ILD: 2 (40) Malignancy: 0 (0)	HCQ + OS: 1 HCQ + OS + immunosuppressants: 2 (1 with MTX, 1 with MTX and MMF) Predisonone + immunosuppressants: 2, (1 with MTX, 1 with AZA and MTX)
Ikeda (47)	Japan	15	Cohort study	Sontheimer criteria	Springerplus	63.0 (60.5–69.0)	10 (66.7)	Heliotrope rash: 8 (53) Gottron's papules: 9 (60)	V-neck sign: 4 (27) Mechanic hands: 2 (13)	Positive ANA: 8 (53)	ILD: 15 (100) Malignancy: 2 (13.3)	OS + CsA + CTX: 6 OS + CsA: 6 OS + CTX: 1 OS: 1 No treatment: 1
Cuesta-Mateos (48)	Spain	11 (8ADM and 3 HDM)	Case series	Modified Sontheimer's definitions	J Eur Acad Dermatol Venereol	56.6 ± 20.6	8 (72.7)	Heliotrope rash: 8 (72.7) Gottron's papules: 10 (90.9)	NA	Positive ANA: 7 (63.6) Elevated CK: 2 (18.2) Myositis in EMG: 4 (36.4) Myositis in muscle biopsy: 0 (0) Skin biopsy consistent with DM: 6 (54.5)	ILD: 1 (9.1) Malignancy: 1 (9.1)	Complete response to treatments: 4 (36.4)
Neri (20)	Italy	8 (2ADM and 6 HDM)	Case series	^&&^	J Clin Neuromuscul Dis	49.3 ± 13.4	6 (75)	NA	NA	Myositis in EMG: 5 (62.5) Myositis in muscle biopsy: 2/2 (100)	ILD: 1 (12.5)	OS + HCQ: 4 OS + MTX: 2 OS + HCQ + CsA:1 OS + HCQ + MTX: 1
Sun (49)	China	41	Case series	Sontheimer criteria	Rheumatol Int	With ILD (*n* = 25): 47.36 ± 10.01, without ILD (*n* = 16): 45.75 ± 14.39	29 (70.7)	Heliotrope rash: 30 (73.2) Gottron's sign: 36 (87.8)	V-neck sign: 23 (56.1)	ANA: 12 (29.3)	ILD: 25 (61) Malignancy: 4 (9.8)	40 patients received systemic steroid (prednisone 0.5–1.0 mg/kg/day or methylprednisolone) combined with immunosuppressive drugs, such as CTX, AZA, or MTX
Sun (50)	China	16	Case-control study	Modified Sontheimer's definitions	Br J Dermatol	45.2 (31–62)	13 (81.3)	Gottron's papules and/or heliotrope rash: 16 (100)	NA	Positive ANA: 3 (18.7) Myositis in EMG or muscle biopsy: 0 (0)	NA	NA
Yamasaki (51)	Japan	21	Case series	Modified Sontheimer's definitions	J Rheumatol	58 ± 16	16 (76)	Typical rash: 21 (100)	NA	Positive ANA: 9 (43)	ILD: 15 (71) Myocarditis: 2 (10)	NA
Azuma (52)	Japan	15	Case series	Sontheimer criteria	Mod Rheumatol	53 ± 19	NA	NA	NA	NA	Malignancy: 3 (20)	7 were treated with immunosuppressive therapy within 6 months of presentation due to progressive ILD
Bendewald (2)	America	6	Case series	Sontheimer criteria	Arch Dermatol	54.3	5 (83.3)	Heliotrope rash: 6 (100) Gottron's papules: 5 (83.3) Gottron's sign: 5 (83.3)	Shawl sign: 5 (83.3) Mechanic hands: 1 (16.7)	Elevated CK: 0 (0) Positive ANA: 6 (100) Elevated ESR: 1/5 (20) Myositis in EMG: 2/5 (40) Myositis in muscle biopsy: 0/0 (0) Muscle inflammation on MRI: 0/1 (0) Skin biopsy consistent with DM: 5/5 (100)	Malignancy: 1 (17) ILD: 1 (16.7, when CADM → CDM)	NA
Cao (16)	China	16 ADM	Case series	Sontheimer criteria	Clin Rheumatol	50.3 ± 16.2	10 (62.5)	Heliotrope rash: 15 (93.8) Gottron's papules: 16 (100) /	NA	Elevated CK with only skin symptoms: 0/16 (0) Positive ANA: 5/16 (31.2) Skin biopsy consistent with DM: 16/16 (100) Myositis in EMG: 0/6 (0) Myositis in muscle biopsy: 0/12 (0) Muscle inflammation MRI: 6/9 (66.7)	ILD: 12 (75) Malignancy: 4 (25)	9 were initially started with prednisone (15–40 mg/day), 2 combination of a reduced dose of cortisone with other immunosuppressive therapy such as MTX or HCQ 4 were treated with HCQ or southernwood in combination with emollients and topical antipruritics 3 who developed ILD started a treatment of MP (80–200 mg/day) combined with MTX
Ye (14)	China	37	Cohort study	Modified Sontheimer's definitions	Clin Rheumatol	With ILD (*n* = 21): 51 ± 9; without ILD (*n* = 7): 48 ± 14	18 (64.3)	NA	NA	Positive ANA: 6/24 (25) Positive MSA: 5/17 (29.4)	ILD: 21/28 (75)	Without ILD: OS <0.5 to 1 mg/kg/day, With ILD: larger dosages OS (≥1 to 2 mg/kg/day) and some undertook MP IV pulse therapy. Most of CADM-ILD patients were treated with a combination of cytotoxic agents, i.e., AZA, CTX, CsA, MMF etc. IVIG was commonly introduced, in addition to other supportive care, but was unsuccessful to those deceased

** *CADM was defined by one of the typical DM rashes without objective muscle weakness for at least 6 months after rash onset and no or minimal abnormalities of serum muscle enzymes (<3 × upper limit of normal), EMG, or muscle biopsy (i.e., minimal histologic changes not significant enough to make a conclusive diagnosis)*.

&& *Presence of DM-specific pathognomonic manifestations—Gottron sign and Gottron papules, heliotrope erythema; Absence of clinically overt muscle involvement for at least 6 months*.

*Modified Sontheimer's definitions (14): (1) ADM: with Gottron's rash or heliotrope rash, but with no symptoms or signs of muscle weakness, normal creatine kinase (CK), electromyography (EMG), and muscle biopsy. (2) HDM: with Gottron's rash or heliotrope rash, but with no symptom of weakness, normal or only mildly reduced muscle strength compatible to age, sex, and severity of systemic illness (should be higher than 4 in the 0–5 grade system). The CK was <1.5 times the upper normal limits. EMG was normal, or only with suspicious myopathic change, i.e., a slight increase of polyphasic potential. The pathological findings were normal or with scant lymphocyte infiltration with normal muscle structure. (3) The CADM is the combination of ADM and HDM. The EMG or biopsy was not available but fulfilled other definitions of ADM/HDM, was treated as possible CADM, and was also included. Duration of the disease is a variant under study, instead of a criterion*.

*ENMC workshop, European Neuromuscular Center international workshop; ILD, interstitial lung disease; OS, oral steroids; MP, methylprednisolone; HCQ, hydroxychloroquine; CTX, cyclophosphamide; MTX, methotrexate; MMF, mycophenolate mofetil; AZA, azathioprine; CsA, Cyclosporin A; IVIG, intravenous immunoglobin; NA, not available*.

Regarding diagnosis, the majority studies used criteria proposed by Sontheimer (5) and Gerami (13). Cao reported that the most common initial diagnoses were contact dermatitis, lichen planus, and seborrheic dermatitis for the 16 patients in their series (16). Patel et al. reported that 73.7% of ADM patients (*n* = 73) in their cohort met the suggested 55% minimum cutoff of 2017 criteria (17). Treatment modalities were introduced in 12 studies. Pinard and researchers from four tertiary care centers evaluated treatments for 115 CADM patients. They found that antimalarials alone were effective in only 11.4% (10 out of 88) and immunosuppressants were given to 80% of whole patients (18). The most commonly used immunosuppressants were MTX (*n* = 59, 51.3%) and MMF or mycophenolic acid (*n* = 46, 40%). In the cohort of Galimberti consisting of 44 CADM patients, prednisone (*n* = 25, 57%) and hydroxychloroquine monotherapy (*n* = 13, 30%) were two commonly used first-line therapy. The majority (64%) required additional medications to control CADM within 6 months of diagnosis despite 29 out of 44 patients had skin improvement with first-line treatment (19).

CADM progressing into classical DM was found in 3 case series. Cao suggested that 3 out of 16 patients (18.75%) developed muscle weakness within 5 years of diagnosis with elevated CK at the time of muscle symptoms (16). Bendewald found that 3 out of 6 CADM patients transformed into classical DM with typical EMG and biopsy findings of DM, 1 of whom developed malignancy (2). Two of eight patients in Neri's series developed clinically evident muscle involvement, 10 months and 6 years after cutaneous disease onset, and were classified as classical DM (20).

## Discussion

The 2017 criteria yielded a sensitivity of 67.2% in our cohort, and the sensitivity of ADM and HDM patients to be classified into IIM were 63.2 and 73.1%, respectively. The most frequently used treatment strategy for these two groups were antimalarial agents alone (31.6%), and oral steroids combined with immunosuppressants (26.9%), respectively. ADM group tended to use more antimalarials (*p* = 0.001, 88.9 vs. 50%) and less steroids (*p* <0.001, 39.1 vs. 92.3%) compared with HDM. The CDASI score improvement was 11.5 (9–17) and 10.5 (8.25–17.25) for ADM and HDM after a median follow-up month of 31.5 (21.75–58) and 32.5 (26.75–57.5), respectively. Six patients developed clinically evident muscle weakness after a median of 10.5 months (IQR 9–13).

The diagnosis of CADM remains a dilemma. A single-center retrospective study showed that 55.6% of confirmed DM cases had a different diagnosis before DM diagnosis, and 80 of 112 (71.4%) in the subgroup of CADM patients had a different diagnosis (21). Lupus and UCTD account for 8.5 and 4.3% of the initial diagnosis, respectively, which is lower than those of the previous reports (4, 21). This discrepancy may be explained by insufficient recognition of immunological skin diseases in primary hospitals and local clinics. While the cutaneous mimickers of DM, such as SLE, could not be differentiated from DM by biopsies, and the distinction between two diseases is mostly anchored by the presentation of pathognomonic cutaneous findings of DM (heliotrope rash, Gottron's) (15).

The 2017 EULAR/ACR criteria proposed a novel classification system of IIM with high sensitivity (93% with muscle biopsies, 87% without muscle biopsies) and high specificity (88% with muscle biopsies, 87% without muscle biopsies) (8). The International Myositis Classification Criteria Project (IMCCP) had a relatively small sample of ADM (*n* = 44) in which 27.3% (*n* = 12) did not meet the cutoff value of IIM (8). 32.8% of CADM patients in our cohort were excluded from the IIM classification tree with a <55% of probability.

Several suggestions may avert the problem of CADM diagnosis, including adding more DM skin items to the 2017 criteria, adjusting score points of the three skin variables in the existing criteria, setting skin biopsy features as a new criterion, and establishing a separate classification system for CADM and skin-symptoms dominant DM (17, 22). To improve the performance of current criteria, some examinations, like EMG, MRI, MSA besides anti-Jo-1 antibody may be considered and correctly assigned in the revised version when applicable (11). Barsotti et al. found that when the variable “dysphasia” was modified into “ILD or dysphasia” of the criteria, six more patients (1.3%) were classified as IIM cases (9).

Following the scheme of skin disease of CADM patients, three levels of treatments were recommended: sun protection, topical treatments and systemic therapy (23). In tradition, the first-line therapy for CADM treatments are antimalarials, however, it may aggravate skin symptoms and may be insufficient to control skin symptoms in moderate to severe cases. Thus, an immunosuppressive agent (e.g., MTX or MMF) is suggested to start alone or as a supplement to hydroxychloroquine (18, 23–25). Of 45 patients (72.6%) treated with hydroxychloroquine in our cohort, 68.8% (31/45) required at least one aggressive therapy for disease control. MTX (*n* = 15, 23.4%) and Tac (*n* = 5, 7.8%) are the most commonly used immunosuppressive agents. In a systematic review of treatments for 153 CADM patients, though 54.9% of patients received antimalarial agents, which was the most common treatment type, 35% stopped this treatment due to inability to wean concomitant steroids or lack of improvement (26).

For patients with refractory or severe cutaneous symptoms, IVIG has been shown to be efficacious and safe for treating skin symptoms. Two relatively large retrospective studies showed that 83–85% of patients exhibited a response to IVIG (27, 28). Emerging therapies such as rituximab, JAK inhibitors (e.g., tofacitinib), and lenabasum can be considered after failure of routine medications (29). Recently, an open-label 12-week study demonstrates strong clinical efficacy of tofacitinib in treating refractory DM as measured by validated myositis response criteria and CDASI (30). The mean score change in CDASI was statistically significant at 12 weeks (28 ± 15.4 at baseline vs. 9.5 ± 8.5 at 12 weeks, *p* = 0.0005).

The subgroup classification of CADM might not be constant during the disease course and might change into classical DM as muscle symptoms developed. Gerami et al. systematically reviewed CADM and identified 37 cases of CADM progressing into classical DM (13%) among 281 subclassified patients. An elevated CK was found in all 14 patients with obtained data at the time of transition to classical DM. Clinically significant muscle symptoms appeared between 15 months and 6 years after onset of characteristic DM skin rash (13). Six patients in our cohort also evolved into classical DM, and the median time to develop muscle weakness was 10.5 months (IQR 9–13). For these patients with suspicious DM, comprehensive tests are recommended at follow-up to exclude disease mimickers and potential systemic involvements. Long-term monitoring for myositis and timely adjusting treatment modalities are essential in the subgroup of CADM.

This study has several limitations. It is a single-center study with relatively small sample size. More wide-scale multicenter studies are needed to improve the generalizability of our findings. Furthermore, the extensive testing of myositis of all cohort members could not be generally measured, and the efficacy of treatment regimens could not be directly compared between groups due to the retrospective nature, which calls for more randomized control studies and case-control studies in the future.

## Conclusions

Our analysis of a population of CADM patients indicated that 32.8% of patients may be overlooked using the three skin variables of 2017 EULAR/ACR criteria. The response rate to single hydroxychloroquine in our cohort was 68.8%, and one aggressive therapy (MTX, Tac, and IVIG) was typically applied with antimalarials for disease control. Clinical characteristics were compared between ADM and HDM. The median of systemic treatments was significantly higher in HDM group. ADM group tended to use more antimalarials (*p* = 0.001, 88.9 vs. 50%) and less steroids (*p* <0.001, 39.1 vs. 92.3%) compared with HDM. The median of CDASI score improvement was 11.5 and 10.5 for ADM and HDM after a median follow-up of 31.5 and 32.5 months, respectively. Six CADM patients had clinically evident muscle weakness after a median of 10.5 months. Long-term monitoring for the development of myositis in patients with CADM, especially those with elevated inflammatory markers at initial visit, might be warranted.

## Data Availability Statement

The original contributions presented in the study are included in the article/Supplementary Material, further inquiries can be directed to the corresponding author/s.

## Ethics Statement

The studies involving human participants were reviewed and approved by the Institutional Board of Peking Union Medical College Hospital. The patients/participants provided their written informed consent to participate in this study.

## Author Contributions

KT reviewed articles, collected data, and wrote the main manuscript text. HZ revised the manuscript. HJ designed the work and critically revised it for important intellectual content. All authors reviewed the manuscript and approved it for publication.

## Funding

This paper was supported by the National Natural Science Foundation of China (81773331 and 82073450) and the National Key Research and Development Program of China (2016YFC0901500).

## Conflict of Interest

The authors declare that the research was conducted in the absence of any commercial or financial relationships that could be construed as a potential conflict of interest.

## Publisher's Note

All claims expressed in this article are solely those of the authors and do not necessarily represent those of their affiliated organizations, or those of the publisher, the editors and the reviewers. Any product that may be evaluated in this article, or claim that may be made by its manufacturer, is not guaranteed or endorsed by the publisher.
