# Supplementary material for: Clinical Characteristics and Management of Patients With Clinical Amyopathic Dermatomyositis: A Retrospective Study of 64 Patients at a Tertiary Dermatology Department
Source: Front Med (Lausanne). 2021 Dec 2;8:783416. doi: 10.3389/fmed.2021.783416 (PMC8674640; doi:10.3389/fmed.2021.783416)
Supplement: Supplementary file 1 [file Data_Sheet_1.docx]

**Supplementary Table 1 Definitions of cutaneous DM terms proposed by Sontheimer and Gerami**

| Terms (Abbreviation) | Definitions |
| --- | --- |
| Amyopathic DM (ADM) | A subset of DM patients characterized by biopsy-confirmed hallmark cutaneous manifestations of classical DM occurring for 6 months or longer with no clinical evidence of proximal muscle weakness and no serum muscle enzyme abnormalities. If more extensive muscle testing is carried out, the results should be within normal limits (if such results are positive or abnormal, the patient can be classified as having “hypomyopathic dermatomyositis”). Exclusion criteria for amyopathic DM include the following:  (1) Treatment with systemic immunosuppressive therapy for 2 consecutive months or longer within the first 6 months after skin disease onset (such therapy could prevent the development of clinically significant myositis).  (2) Use of drugs at the time of cutaneous disease onset that are known to be capable of producing isolated DM-like skin changes (eg, hydroxyurea) |
| Hypomyopathic DM (HDM) | A designation for patients with cutaneous DM and no clinical evidence of muscle disease (ie, weakness) for 6 months or longer, who during evaluation are found to have subclinical evidence of myositis upon laboratory (eg, muscle enzymes), electrophysiologic, and/or radiologic evaluation. The same exclusion criteria listed for amyopathic DM above apply here as well. |
| Clinically amyopathic DM (CADM) | A functional umbrella designation used to refer to amyopathic DM and/or hypomyopathic DM patients as defined here (ie, clinically amyopathic DM = amyopathic DM + hypomyopathic DM). The CADM designation has been coined to emphasize the fact that the predominant clinical problem is protracted skin disease in patients so affected. |
| Clinically amyopathic DM evolving into classical DM (CADM → CDM) | A working designation for those individuals in whom the onset of clinically significant muscle disease (ie, weakness) appears greater than 6 months after an initial disease presentation as CADM. |

**Supplementary Table 2 Predictive value of muscle enzymes for extensive muscle testing (EMG and muscle biopsy) among our ADM and HDM patients**

|  | Typical muscle biopsy or EMG finding | Normal or minor biopsy and EMG finding |  |
| --- | --- | --- | --- |
| Elevated muscle enzymes | 6 | 11 | Positive predictive value = 54.5% |
| Normal muscle enzymes | 4 | 25 | Negative predictive value = 86.2% |

**Supplementary Table 3 Clinical characteristics of the patients with hypoamyopathic dermatomyositis evolving into classical DM**

|  | CADM → classical DM |
| --- | --- |
| Patients (n/%) | 6 (9.4) |
| Demographics |  |
| Female sex | 5 (76.3) |
| Age at disease diagnosis (years) | 44.8±19.6 |
| Time to diagnosis (mons) | 5.5±4.5 |
| CDASI score |  |
| CDASI score at initial visit | 5.8±3.4 |
| a-score | 5.5±3.5 |
| d-score | 0.3±0.5 |
| CDASI score at last visit | 3.2±2.8 |
| a-score | 2.3±2.3 |
| d-score | 0.8±0.8 |
| Examinations |  |
| Elevated serum muscle enzymes (CK, LDH, AST) | 3/6 (83.3) |
| Elevated Inflammatory markers (ESR, hsCRP) | 3/6 (50) |
| Positive ANA (≥1:80) | 3/6 (50) |
| Positive MSA | 2/2 (100) |
| Myogenic lesions in EMG | 2/3 (80) |
| Muscle inflammation on MRI | 4/4 (100) |
| Treatments |  |
| Treatments prior to development of muscle weakness | 2 (2-2.5) |
| Systemic treatments | 2 (1.5-2) |
| Prednisone | 3 (50) |
| Antimalarials | 4 (66.7) |
| Immunosuppressants | 1 (16.7) |
| Topical treatments | 4 (66.7) |
| Total treatments | 4 (3-5) |
| Systemic treatments | 3 (2-4) |
| Prednisone | 6 (100) |
| Antimalarials | 4 (66.7) |
| Immunosuppressants | 4 (66.7) |
| Topical treatments | 6 (100) |
| Outcomes |  |
| Median follow-up (mons) | 38 (27.5-69) |
| Clinical remission of skin disease | 4 (66.6) |

**Supplementary Table 4 Univariate analysis of risk factors associated with cancer, interstitial lung disease and muscle weakness development for dermatomyositis patients**

|  | OR | 95%CI | P-value |
| --- | --- | --- | --- |
| Cancer |  |  |  |
| Age> 50 years | 3.30 | 0.70-15.48 | 0.13 |
| Male sex | 2.05 | 0.41-10.14 | 0.38 |
| Elevated Inflammatory markers | 3.05 | 0.56-16.52 | 0.20 |
| Positive ANA | 4.94 | 0.89-27.39 | 0.07 |
| Interstitial lung disease |  |  |  |
| Age> 50 years | 2.68 | 0.89-8.10 | 0.08 |
| Male sex | 0.80 | 0.24-2.73 | 0.72 |
| Elevated Inflammatory markers | 2.52 | 0.62-10.26 | 0.20 |
| Positive ANA | 0.83 | 0.27-2.51 | 0.74 |
| Muscle weakness development |  |  |  |
| Age> 50 years | 0.79 | 0.10-9.81 | 0.98 |
| Male sex | 0.68 | 0.04-11.52 | 1.47 |
| Elevated Inflammatory markers | 0.08 | 0.01-0.61 | **0.02** |
| Positive ANA | 0.46 | 0.05-3.81 | 0.74 |

**Supplementary Table 5 Performance of 2017 European League Against Rheumatism/American College of Rheumatology (EULAR/ACR) criteria**

| Probability of IIM | Classification of IIM | ADM | HDM | p-value |
| --- | --- | --- | --- | --- |
| ＜50% | Excluded | 13 (31.6) | 3 (11.5) | 0.076 |
| 50%≤~＜55% | Possible | 0 (0) | 3 (11.5) | 0.062 |
| 55%≤~＜90% | Probable | 13 (34.2) | 6 (26.9) | 0.593 |
| ≥ 90% | Definite | 12 (31.6) | 14 (53.8) | 0.193 |

**Supplementary Table 6 Presence of the three characteristic skin variables proposed by 2017 EULAR/ACR criteria in patients with ADM and HDM at the time of enrollment**

| **ADM (n=38)** | **Heliotrope rash** | **Gottron’s papules** | **Gottron’s sign** | 7 (10.9) |
| --- | --- | --- | --- | --- |
|  |  |  | **No Gottron’s sign** | 9 (14.1) |
|  |  | **No Gottron’s papules** | **Gottron’s sign** | 6 (9.4) |
|  |  |  | **No Gottron’s sign** | 9 (14.1) |
|  | **No heliotrope rash** | **Gottron’s papules** | **Gottron’s sign** | 3 (4.7) |
|  |  |  | **No Gottron’s sign** | 2 (3.1) |
|  |  | **No Gottron’s papules** | **Gottron’s sign** | 1 (1.6) |
|  |  |  | **No Gottron’s sign** | 1 (1.6) |
| **HDM (n=26)** | **Heliotrope rash** | **Gottron’s papules** | **Gottron’s sign** | 5 (7.8) |
|  |  |  | **No Gottron’s sign** | 3 (4.7) |
|  |  | **No Gottron’s papules** | **Gottron’s sign** | 4 (6.3) |
|  |  |  | **No Gottron’s sign** | 6 (9.4) |
|  | **No heliotrope rash** | **Gottron’s papules** | **Gottron’s sign** | 2 (3.1) |
|  |  |  | **No Gottron’s sign** | 2 (3.1) |
|  |  | **No Gottron’s papules** | **Gottron’s sign** | 1 (1.6) |
|  |  |  | **No Gottron’s sign** | 3 (4.7) |
